# Media multitasking, depression, and anxiety of college students: Serial mediating effects of attention control and negative information attentional bias

**DOI:** 10.3389/fpsyt.2022.989201

**Published:** 2022-08-18

**Authors:** Shiyi Li, Lifang Fan

**Affiliations:** ^1^Key Research Base of Humanities and Social Sciences of the Ministry of Education, Academy of Psychology and Behavior, Tianjin Normal University, Tianjin, China; ^2^Faculty of Psychology, Tianjin Normal University, Tianjin, China; ^3^Tianjin Social Science Laboratory of Students’ Mental Development and Learning, Tianjin, China

**Keywords:** media multitasking, attentional control, attention bias, depression, anxiety

## Abstract

**Background:**

The COVID-19 epidemic provides an environment for frequent media multitasking, which might associate with an increase in depression and anxiety. Since many studies have found that media multitasking negatively affects cognitive capacity, we propose a cognitive perspective to explore how media multitasking may associate with mental health. This study examined the potential mediating role of attention control and negative information attentional bias in the relationship between media multitasking and anxiety and depression.

**Methods:**

Participants (*n* = 567) were recruited from college students in China. They completed an online survey that included the Media Multitasking Inventory (MMI), Attention Control Scale (ACS), Attention to Positive and Negative Information Scale (APNI), Generalized Anxiety Disorder Scale (GAD-7), and Patient Health Questionnaire (PHQ-9). After exploring the correlations between the measures, serial mediation models were examined.

**Results:**

The results indicated significant positive correlations between media multitasking and anxiety and depression. Media multitasking, anxiety, and depression were negatively correlated with attention focusing, while positively correlated with negative information attention bias. Media multitasking did not correlate with attention shifting. Mediation modeling demonstrated that attention focusing and negative information attention bias played a serial mediating role in the relationship between media multitasking and anxiety and depression. However, the results did not support the serial mediation model through attention shifting and negative information attention bias.

**Conclusion:**

Media multitasking does not directly influence anxiety and depression, while attention focusing and negative information attention bias play serial mediating roles in their relationship. This study highlights the potential cognitive mechanisms between media multitasking and anxiety and depression, providing theoretical support for interventions in individual mental health during the epidemic.

## Introduction

The global mental health report released by the WHO recently showed that depression and anxiety increased by 25% globally in 2020, the year of the COVID-19 epidemic outbreak (1). The development of technology has allowed for the high accessibility and portability of media devices, which facilitated working and studying at home during the pandemic.

Media multitasking is typically known as simultaneously engaging in multiple media tasks, such as checking messages on the cellphone while watching TV or reading a book while listening to music (2, 3). The convenience of mobile devices makes it possible to do multiple media activities simultaneously. Surveys have shown that when they use media, people spend 25–50% of their time consuming multiple media simultaneously (4, 5), and this number is continuously growing (6). It is important to notice that numerous studies showed that media multitasking behavior could detrimentally affect cognitive ability, such as attention and memory (7–9). Other studies have even found that media multitasking poses a potential threat to the mental health of the public (10, 11).

Several studies suggested that frequent media multitasking behaviors may hurt mental health, possibly leading to anxiety and depression (12, 13). Becker et al. proposed that media multitasking can predict the levels of depression and social anxiety of college students, even under the control of other factors (such as total time spent on media and personality traits) (14). At the same time, there has been little research on how media multitasking affects anxiety and depression, most of which prioritized the mediating role of personality traits or environmental factors such as peer relationships and stress (15, 16). For example, Shin et al. (17) proposed that media multitasking may serve as an avoidance-oriented behavioral coping strategy to divert attention from unpleasant information. Long-term avoidance behavior is not beneficial for acquiring adaptive coping strategies (i.e., problem-solving); thus, frequent media multitasking over time may lead to greater susceptibility to anxiety and depression (18). However, it has been suggested that cognition plays a mediating role between behavior and emotion (19, 20). For example, a study found that mindfulness moderated the relationships between mobile phone addiction and anxiety and depression (21). However, the cognitive process by which media multitasking negatively affects anxiety and depression is not clear.

Negative information attention bias refers to a tendency to attend to threatening or negative stimuli compared to neutral stimuli (22). More and more evidence shows that negative information attention bias is not only a phenomenon or symptom accompanying some psychological disorders, but also a central cognitive factor in their development, maintenance, and recurrence (23, 24). Attention bias to negative stimuli could result in anxiety and depression (25, 26). Cret performed attentional bias modification training (ABMT) on participants, and the results showed that participants in the negative ABMT condition had higher levels of anxiety than before the training, suggesting a causal link between attentional bias toward emotional information and anxiety (27). Krejtz et al. also confirmed that the depressive symptoms of depressed patients could be effectively reduced by changing negative information attentional bias (28). In addition, a previous study found that ordinary individuals will be attracted to negative information while media multitasking and the negative information elicited more significant unpleasantness (29). All those evidence supports the hypothesis that negative information attention mediates the association between media multitasking and anxiety and depression.

Why do some people show a cognitive processing pattern of negative information attention bias? Some studies suggest it may be due to reduced attentional control ability (27, 30). Attentional control refers to top-down flexible regulation of attentional resources, involving allocating attention in the face of competing or conflicting demands (31). According to attention control theory (30, 32), the negative information attention bias of anxious and depressed individuals is a cognitive deficit dependent on attentional control. The theory considers that anxiety and depression disrupt the balance between the goal-directed attentional system (top-down control) and the stimulus-driven attentional system (bottom-up control). So, with a higher level of anxiety or depression, people may prioritize allocating attentional resources to the negative stimulus, which in turn increases the level of anxiety and depression, resulting in a vicious circle. Some studies have found that higher attentional control facilitates people to recruit cognitive resources to inhibit unintentional attention to negative stimuli in a top-down way, whereas lower attentional control predisposes a person to over-preference for negative stimuli (33, 34). Other studies also found that attentional control may be an essential protective factor for mental health. People with valid attentional control can avoid negative thoughts, coping styles, and emotional reactions, thereby maintaining a lower level of anxiety or depression (17, 35). Therefore, attentional control may influence attentional bias and lead to anxiety and depression.

In the past decades, many studies found that media multitasking is associated with poor cognitive functioning. Notably, it leads to a reduced attentional control ability (36, 37). Attentional control ability has two aspects: attention focusing (the ability to maintain attentional engagement when facing distraction) and attention shifting (the ability to switch between different tasks or shift attention from distractions to new or related tasks) (33). For attention focusing, Ophir et al. initially found that heavy media multitaskers are more liable to fail when they need to filter distractions (2). This finding is proved by many subsequent studies (38, 39). However, the relationship between media multitasking and attention shifting is mixed. Some researchers found that heavy media multitaskers alternate between two different tasks with more difficulty and pay higher shifting costs (40, 41). At the same time, other studies indicated that heavy media multitaskers are more efficient with task-shifting (42, 43). In brief, the negative effect of frequent media multitasking on attention focusing was confirmed by multiple studies, whereas the results about attention shifting were inconsistent. Thus, we will consider the two aspects separately in the present study.

**FIGURE 1:**
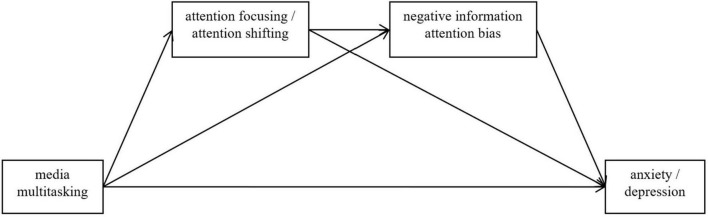
The mediating pathway of attention focusing/shifting and negative information attention bias in media multitasking influence anxiety/depression.

Based on the above literature review, it is reasonable to conclude that frequent media multitasking behavior may lead to poor attention control. Consequently, it forms the negative information attention bias, eventually leading to the occurrence or exacerbation of anxiety or depression symptoms. Thereby, the present research will test the hypotheses below. (1) Media multitasking is positively correlated with anxiety and depression; (2) Media multitasking, anxiety, and depression have a significant negative association with attentional control (including attention focusing and attention shifting) and a significant positive association with negative information attention bias; (3) Attentional control (separated as attention focusing and attention shifting) and negative information attention bias play a serial mediating role in the relationship between media multitasking, anxiety, and depression (see Figure 1). This study has potential significance for understanding the relationship between media multitasking and mental health from a cognitive perspective. This understanding will be used to prevent potential mental illness induced by media multitasking during the COVID-19 pandemic.

## Methods

### Participants and procedure

A cross-sectional study was conducted on an online survey tool, Wen Juan Website.^1^ We recruited all participants *via* WeChat (a popular Chinese social media platform). College students from one university usually have one joint WeChat group. Several students from Tianjin Normal University initially sent the recruitment messages to WeChat groups of students. The snowball sampling method was adopted to increase the sample size. We encouraged participants who saw and joined our study to share the link with more college students. To avoid data duplication, each IP address was only granted access to the survey once. Also, we identify the location of the participants *via* IP address. Finally, we obtained samples from 28 provinces in China, most of which were from Tianjin, Fujian, Sichuan, and Beijing.

All subjects participated voluntarily and were informed that the survey was anonymous and confidential. The study obtained the electrical consent of all subjects. All procedures in this study met the ethical standards of the Chinese Psychological Association^2^ and conformed to the 1964 Declaration of Helsinki and subsequent amendments or similar ethical standards. The Ethics Committee of Tianjin Normal University approved the study (022050901).

A total of 617 students participated and completed the survey. After excluding unqualified samples (e.g., some participants completed the questionnaire battery in <180 s or >15 min), we finally collected 567 valid participants with an effective response rate of 91.90%. The participants were 21.4 years old on average (SD = 2.24 years), with 241 males (42.50%) and 326 females (57.50%). Most participants were undergraduates (*n* = 501, 88.36%), and the remaining were postgraduate students.

### Measures

#### Media multitasking inventory

The Media Multitasking Inventory (MMI) was initially developed by Ophir et al. (2) and developed by Madore et al. (8). In this study, the MMI was modified from Madore et al. The questionnaire includes two parts. In part 1, participants were instructed to report the total number of hours per week typically spent doing each of eight media activities: self-regulated learning (doing homework, self-study, writing papers), reading (novels, comics, etc.), taking phone calls or video calls, playing video games, watching videos (watching TV, movies, online videos, etc.), listening to music, browsing the Internet (news web and other non-social websites), using social media applications (chatting online with WeChat, posting or browsing on Weibo, etc.). In part 2, participants indicated how often they simultaneously engaged in each of the other activities while doing the primary media activities on a four-point Likert scale [never (0), occasionally (0.33), often (0.67), always (1)]. MMI index is calculated as an indication of the level of media multitasking the participant engaged in during a typical media-consumption hour (2). MMI is designed to measure the frequency of specific media multitasking behaviors without further inferences about possible latent variables. Thus, it is a valid index for the media multitasking activity (9).

#### The attention to positive and negative information scale

The Attention to Positive and Negative Information Scale (APNI) was developed by Noguchi et al. to measure the attention bias of individuals toward negative or positive information, and the revised Chinese version was used in this study (44, 45). The scale includes 30 items in total, which is divided into two dimensions: positive information attention bias (19 items) and negative information attention bias (11 items). Items are rated on a 5-point Likert scale from 1 (“strongly disagree”) to 5 (“strongly agree”). Only the negative attention bias dimension was used in this study (APNI-N). Examples of the items in APNI-N are: “I don’t forget when others do things that hurt me,” “I pay special attention to bad news on the television news.” The Cronbach’s alpha for this sample was 0.91.

#### The attentional control scale

The Attentional Control Scale (ACS) was developed by Derryberry et al. to measure a general capacity for attentional control, and the revised Chinese version was used in this study (33, 46). The scale is divided into two dimensions: attention focusing (8 items, e.g., “When concentrating, I can focus my attention so that I become unaware of what’s going on in the room around me,” “My concentration is good even if there is music in the room around me”) and attention shifting (8 items, e.g., “It is easy for me to alternate between two different tasks,” “I can quickly switch from one task to another”), for a total of 16 items. Each item is rated on a 4-point Likert scale from 1 (“almost never”) to 4 (“always”). Possible scores range from 16 to 64, with higher scores indicating a greater capacity for attentional control. The Cronbach’s alpha for this sample was 0.81.

#### Generalized anxiety disorder questionnaire-7

The Generalized Anxiety Disorder Questionnaire (GAD-7) was developed to assess the defining symptoms of GAD in the last 2 weeks (47). There are seven items rated on a 4-point Likert scale from 0 (“not at all”) to 3 (“nearly every day”). Examples of the items are: “Feeling nervous, anxious or on edge,” “Not being able to stop or control worrying.” Scores range from 0 to 21, with higher scores indicating more severe anxiety symptoms. The Cronbach’s alpha of this scale in this sample was 0.90.

#### Patient health questionnaire-9

The Patient Health Questionnaire-9 (PHQ-9) was used as a self-administered screening tool for assessing the severity of depressive symptoms. PHQ-9 includes nine items based on the Diagnostic and Statistical Manual of Mental Disorders, 4th Edition (DSM-IV) for depression (48). The questionnaire was measured by participants reporting their mood during the immediately preceding 2 weeks. Each item was scored on a 4-point Likert scale from 0 (“not at all”) to 3 (“nearly every day”). Examples of the items are: “Feeling down, depressed, or hopeless,” “Little interest or pleasure in doing things.” Scores range from 0 to 27, with higher scores indicating more severe depression symptoms. The Cronbach’s alpha of this scale in this sample was 0.92.

### Data analysis

Data analyses were conducted by the IBM SPSS Statistics for Windows, version 26.0 (IBMCorp., Armonk, NY, United States). First, descriptive analyses were conducted for the variables of interest for the total sample. Then Harman’s single-factor test was conducted to examine the common method bias. Pearson correlation was used to examine the correlations among variables. This study used deviation-corrected percentile bootstrapping to test. To test the significance of the indirect effect using the Hayes Process Macro (model six) for SPSS with a 95% bias-corrected confidence interval (CI) based on 5,000 bootstrap samples. A significant mediation was determined if the CI around the indirect effect did not include 0.

Harman’s single-factor test was used to test for common method bias (49). The results of unrotated factor analysis showed that seventeen factors with eigenvalues greater than one emerged and accounted for 65.23% of the total variance. The first principal factor explained 30.30% of the variance (<40%). Therefore, it indicated that common method bias was not a concern in this study.

## Results

### Descriptive statistics and correlations

The Descriptive statistics and Pearson correlation results are shown in Table 1. Specifically, media multitasking was significantly positively correlated with anxiety (*r* = 0.195, *p* < 0.01) and depression (*r* = 0.221, *p* < 0.01) and negative information attention bias (*r* = 0.358, *p* < 0.01), but significantly negatively correlated with attention focusing (*r* = −0.303, *p* < 0.01), and not correlated with attention shifting. Moreover, attention focusing, attention shifting was significantly and negatively correlated negative information attention bias (*r* = −0.535, *p* < 0.01; *r* = −0.358, *p* < 0.01), anxiety (*r* = −0.390, *p* < 0.01; *r* = −0.300, *p* < 0.01), and depression (*r* = −0.410, *p* < 0.01; *r* = −0.331, *p* < 0.01). Negative information attention bias was significantly and positively correlated with anxiety (*r* = 0.521, *p* < 0.01) and depression (*r* = 0.515, *p* < 0.01). The results showed that subjects with more likeness to engage in media multitasking had lower levels of attention focusing and more attention bias toward negative information, thus having higher anxiety and depression scores.

**TABLE 1:** Mean, standard deviation, and correlation coefficient of each variable.

	M	SD	1	2	3	4	5	6
1. MMI	2.83	1.65						
2. AF	20.78	4.55	−0.303**					
3. AS	20.77	3.69	−0.076	0.602**				
4. ACS	41.56	7.39	−0.225**	0.917**	0.871**			
5. APNI-N	33.64	9.81	0.358**	−0.535**	−0.358**	−0.508**		
6. GAD-7	4.93	4.32	0.195**	−0.390**	−0.300**	−0.390**	0.521**	
7. PHQ-9	6.93	5.18	0.221**	−0.410**	−0.331**	−0.418**	0.515**	0.784**

MMI, Media Multitasking Inventory; AF, Attention focusing dimension of Attentional Control Scale; AS, Attention shifting dimension of Attentional Control Scale; ACS, Attentional Control Scale; APNI-N, Negative dimension of the Attention to Positive and Negative Information Scale; GAD-7, Generalized Anxiety Disorder Questionnaire-7; PHQ-9, Patient Health Questionnaire-9. **p < 0.01.

### The serial mediating analysis

#### Media multitasking-anxiety serial mediated analysis

Attention focusing and attention shifting are separated as two independent dimensions to create the mediation model. Multiple mediation analysis was conducted with media multitasking as the independent variable, attention focusing and negative information attention bias as mediating variables, anxiety as the dependent variable, and gender and age as covariates. The models with attention focusing and negative information attention bias as mediating variables were significant. The results are shown in Table 2. Results indicate that Media multitasking cannot significantly predict anxiety (β = −0.026, *p* = 0.515), but significantly predict attention focusing and negative information attention bias (β = −0.299, *p* < 0.001; β = 0.212, *p* < 0.001). In addition, attention focusing can significantly predict negative information attention bias (β = −0.469, *p* < 0.001), anxiety was significantly predicted by attention focusing and negative information attention bias (β = −0.157, *p* < 0.001; β = 0.441, *p* < 0.001).

**TABLE 2:** Regression analysis of variable relationships in models.

Outcome variable	Predictor variables	*R*	*R* ^2^	*F*	β	*t*
AF		0.304	0.093	19.150***		
	Gender				0.012	0.141
	Age				−0.023	−0.569
	MMI				−0.299	−7.139***
APNI-N		0.574	0.329	68.977***		
	Gender				−0.007	−0.103
	Age				0.024	0.688
	MMI				0.212	5.634***
	AF				−0.469	−12.938***
GAD-7		0.542	0.293	46.552***		
	Gender				−0.132	−1.762
	Age				−0.026	−0.729
	MMI				−0.026	−0.651
	AF				−0.157	−3.696***
	APNI-N				0.441	10.181***

MMI, Media Multitasking Inventory; AF, Attention focusing dimension of Attentional Control Scale; APNI-N, Negative dimension of the Attention to Positive and Negative Information Scale, GAD-7, Generalized Anxiety Disorder Questionnaire - 7. ***p < 0.001.

**FIGURE 2:**
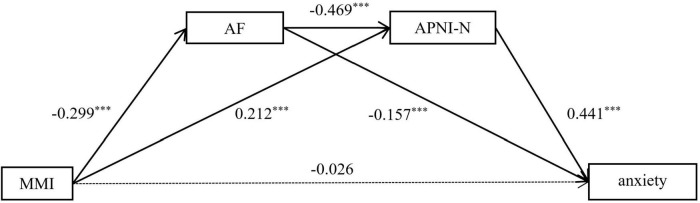
The mediating pathway of attention focusing and negative information attention bias in media multitasking influence anxiety. MMI, Media Multitasking Inventory; AF, Attention Focusing dimension of Attentional Control Scale; APNI-N, Negative dimension of the Attention to Positive and Negative Information Scale. ^***^p < 0.001.

Then we performed a bootstrap analysis using the bias correction non-parametric percentage test to further examine the serial mediating effects. The results revealed (see Table 3) that the direct effect of media multitasking on anxiety was not significant (*p* = 0.515) and that attention focusing and negative information attention bias mediated the relationship between media multitasking and anxiety. Specifically, this mediating effect consisted of three pathways, namely indirect pathway 1: media multitasking → attention focusing → anxiety; indirect pathway 2: media multitasking → negative information attention bias → anxiety; indirect pathway 3: media multitasking → attention focusing →negative information attention bias → anxiety. The effect values of the three pathways were 0.266, 0.531, and 0.349, respectively. The 95% confidence interval of the three paths did not contain 0, indicating that the serial mediation effect was significant (pathway model see Figure 2).

**TABLE 3:** Mediating paths between media multitasking and anxiety.

	Effect	Boot SE	Boot LLCI	Boot ULCI	Relative effect (%)
Total	0.177				
Total indirect effect	0.203	0.026	0.153	0.255	1.147
Indirect effect 1	0.047	0.014	0.020	0.077	0.266
Indirect effect 2	0.094	0.019	0.057	0.131	0.531
Indirect effect 3	0.062	0.011	0.041	0.085	0.349

Relative effect (%) = Indirect effect/Total; Indirect effect 1: media multitasking → attention focusing → anxiety; Indirect effect 2: media multitasking → negative information attention bias → anxiety; Indirect effect 3: media multitasking → attention focusing → negative information attention bias → anxiety.

The models with attention shifting and negative information attention bias as mediating variables were not significant. Results indicate that Media multitasking did not significantly predict anxiety and attention shifting (β = 0.001, *p* = 0.979; β = −0.081, *p* = 0.067), but media multitasking significantly and positively predicted negative information attention bias (β = 0.326, *p* < 0.001). Moreover, attention shifting significantly predicted negative information attention bias (β = −0.332, *p* < 0.001), and attention shifting and negative information attention bias significantly predicted anxiety (β = −0.133, *p* < 0.001, β = 0.468, *p* < 0.001). It can be seen that the path coefficient of media multitasking to attentional shifting was not significant. The results indicate that attention shifting and negative information attention bias had no significant serial mediating effect between media multitasking and anxiety.

#### Media multitasking–depression serial mediated analysis

Next, the study analyzed the mediating effect of attention focusing and negative information attention bias between media multitasking and depression. Multiple mediation analysis was conducted with media multitasking as the independent variable, attention focusing and negative information attention bias as mediating variables, depression as the dependent variable, and gender and age as covariates. The models with attention focusing and negative information attention bias as mediating variables were significant. The results are shown in Table 4. Results indicate that Media multitasking did not significantly predict depression (β = −0.004, *p* = 0.913), but significantly predicted attention focusing and negative information attention bias (β = −0.299, *p* < 0.001, β = 0.212, *p* < 0.001). In addition, attention focusing were negatively significantly predicted negative information attention bias (β = −0.469, *p* < 0.001), and depression significantly predicted by attention focusing and negative information attention bias (β = −0.184, *p* < 0.001, β = 0.411, *p* < 0.001).

**TABLE 4:** Regression analysis of variable relationships in models.

Outcome variable	Predictor variables	*R*	*R* ^2^	*F*	β	*t*
AF		0.304	0.093	19.150***		
	Gender				0.012	0.141
	Age				−0.023	−0.569
	MMI				−0.299	−7.139***
APNI-N		0.574	0.329	68.977***		
	Gender				−0.007	−0.103
	Age				0.024	0.688
	MMI				0.212	5.634***
	AF				−0.469	−12.938***
PHQ-9		0.548	0.300	48.136***		
	Gender				−0.192	−2.577
	Age				−0.037	−1.050
	MMI				−0.004	−0.109
	AF				−0.184	−4.367***
	APNI-N				0.411	9.522***

MMI, Media Multitasking Inventory; AF, Attention focusing dimension of Attentional Control Scale; APNI-N, Negative dimension of the Attention to Positive and Negative Information Scale; PHQ-9, Patient Health Questionnaire-9. ***p < 0.001.

**FIGURE 3:**
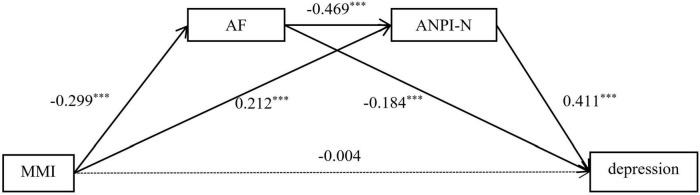
The mediating pathway of attention focusing and negative information attention bias in media multitasking influence depression. MMI, Media Multitasking Inventory; AF, Attention Focusing dimension of Attentional Control Scale; APNI-N, Negative dimension of the Attention to Positive and Negative Information Scale. ^***^p < 0.001.

Then we performed a bootstrap analysis using the bias correction non-parametric percentage test to further examine the significance of the serial mediating effects. The results revealed (see Table 5) that the direct effect of media multitasking on depression was not significant (*p* = 0.913) and that attention focusing and negative information attention bias mediated the relationship between media multitasking and depression. Specifically, this mediating effect consisted of three pathways, namely indirect pathway 1: media multitasking → attention focusing → depression; indirect pathway 2: media multitasking → negative information attention bias → depression; indirect pathway 3: media multitasking → attention focusing → negative information attention bias → depression. The effect values of the three pathways were 0.281, 0.444, and 0.296, respectively. The 95% confidence interval of the three paths did not contain 0, indicating that the serial mediation effect was significant (pathway model see Figure 3).

**TABLE 5:** Mediating paths between Media multitasking and depression.

	Effect	Boot SE	Boot LLCI	Boot ULCI	Relative effect (%)
Total	0.196				
Total indirect effect	0.200	0.024	0.152	0.247	1.020
Indirect effect 1	0.055	0.014	0.029	0.084	0.281
Indirect effect 2	0.087	0.018	0.055	0.123	0.444
Indirect effect 3	0.058	0.011	0.038	0.080	0.296

Relative effect (%) = Indirect effect/Total; Indirect effect 1: media multitasking → attention focusing → depression; Indirect effect 2: media multitasking → negative information attention bias → depression; Indirect effect 3: media multitasking → attention focusing → negative information attention bias → depression.

The models with attention shifting and negative information attention bias as mediating variables were not significant. Results indicate that media multitasking cannot significantly predicted depression and attention shifting (β = 0.028, *p* = 0.471; β = −0.081, *p* = 0.067), but media multitasking significantly and positively predicted negative information attention bias (β = 0.326, *p* < 0.001). Moreover, attention shifting significantly predicted negative information attention bias (β = −0.332, *p* < 0.001), and depression significantly predicted by attention shifting and negative information attention bias (β = −0.174, *p* < 0.001; β = 0.435, *p* < 0.001). It can be seen that the path coefficient of media multitasking to attentional shifting was not significant. The results indicate that attention shifting and negative information attention bias had no significant serial mediating effect between media multitasking and depression.

## Discussion

Our study aimed to explore the function of attention-related cognitive factors while media multitasking influences individuals’ anxiety and depression symptoms. In this study, media multitasking was significantly and positively related to anxiety and depression, and hypothesis (1) was verified; media multitasking, anxiety, and depression were significantly negatively related to attention focusing, significantly positively related to negative information attention bias, but media multitasking was not significantly related to attentional shifting, and hypothesis (2) was partially verified; attention focusing and negative information attention bias played a serial mediating role in the relationship between media multitasking, anxiety, and depression, but the serial mediating effect of media multitasking-attention shifting-negative information attention bias-anxiety/depression is not significant, and hypothesis (3) was partially verified.

The results show that frequent media multitasking behavior is associated with higher levels of anxiety and depression, which are consistent with previous research (12, 14). A more important finding of this study was that after adding two mediating variables to the model, the direct effect of media multitasking on anxiety or depression was not significant, while the three indirect effects were significant, and the two cognitive factors played a serial mediating role. These results suggest that media multitasking may not directly influence anxiety and depression, but when considering attention focusing and negative information attention bias as the mediators, the serial effect is significant. The mediating effect found in this study could explain why some studies have found that media multitasking has no implications for mental health (50).

The present study found a significant positive association between media multitasking and negative information attention bias, and a significant positive association between negative attention bias and anxiety and depression. Negative information attention bias can partially mediate the relationship between media multitasking and anxiety and depression. As in a previous study, researchers simulated real-world media multitasking by asking participants to watch television news while paying attention to occasional tweets popping up on a tablet, the result showed that participants looked at negative tweets longer than at the positive ones, and participants tended to have more negative feelings (29). During the COVID-19 epidemic, learning and working remotely at home is becoming regular and promoting more media multitasking behaviors. Media multitasking may expose people to more negative or positive information on the Internet and induce greater negative emotions such as anxiety and depression if they have an attentional bias toward negative information (26, 51).

Furthermore, the present study found that attention focusing and negative information attentional bias played a serial mediating role in the relationship between media multitasking, anxiety, and depression. The more frequently individuals engaged in media multitasking had more significant decreases in attentional control and were more likely to attend to negative information, leading to increased levels of anxiety and depression. This is consistent with previous studies, which found that heavy media multitaskers perform worse in attentional control than light media multitaskers (2, 52). Previous study has also found that individuals with higher attentional control ability have lower levels of anxiety and depression (53). Therefore, attentional control may be a protective mechanism for the mediating role of negative attention bias mediating the relationship between media multitasking and anxiety and depression. Individuals with good attentional control ability can regulate top-down attention allocation and avoid bottom-up stimulus drive systems that are overly enhanced and more easily attracted to negative stimuli. However, the lower the attentional control, the more susceptible the individual is to the stimulus drive system, and the more attention is captured by negative stimuli, leading to anxiety and depression (54–56).

The two dimensions of attentional control were analyzed separately in this study, and the results indicated that different aspects of attentional control did not play the same mediating role. Remarkably, the serial mediation of attention focusing and negative information attentional bias was held, but the serial mediation of attention shifting and negative attentional bias was not. The above results suggest that media multitasking has different effects on attention focusing and attention shifting. A more consistent finding from previous studies revealed that media multitasking harmed attention focusing. For example, heavy media multitaskers tend to use breadth-biased attention allocation, are more likely to be inattentive, and get distracted by internal or external irrelevant stimuli (39). While the results of studies on the influence of media multitasking on attention shifting are inconsistent, some studies suggested that heavy media multitasker’s switch cost was greater than light media multitaskers on shifting tasks (40, 41). However, others have found that heavy media multitaskers behave better when shifting among tasks (43, 57). The discrepancy in the results of these studies may be due to the different effects of different media multitasking types on attention. Which previous scholars suggested can be defined as two different behaviors: simultaneous media multitasking and media task-shifting. It has been suggested that different types of media multitasking may have different effects on attention shifting (6, 58). The reason why media multitasking cannot insignificantly predict attention shifting in the present study may be that different types of media multitasking were not distinguished.

In this electronic age, mobile devices (PCs, tablets, smartphones, etc.) have become an essential part of our lives, which makes it hard to avoid the increase in media multitasking. An important practical implication of the present study is that the cognitive decline resulting from media multitasking may induce mental health problems. Several studies have proved the effeteness of attentional control training (59, 60). Thus, improving the attention control ability of heavy media multitaskers could be a possible way to prevent the harmful effect.

## Limitation and prospects

The current study has several limitations. Firstly, because our data are cross-sectional, we cannot establish evidence of a causal relationship between media multitasking and anxiety and depression. Longitudinal designs should be considered in future research to test the causality. Secondly, the self-report method could introduce response bias, such as overestimating or underestimating their media use time and cognitive abilities. Further studies are needed to develop more objective methods to measure the occurrences of media multitasking, such as the experience sampling approach (61). Alternatively, observing the real-time changes in cognitive abilities and emotions in the lab could be considered in future studies. Finally, as recent research suggested, the different patterns of media multitasking could play a different role (3, 6). Future research should focus on the effect of subdividing media multitasking types on attention control and mental health.

## Conclusion

In conclusion, this study found that media multitasking was significantly associated with anxiety and depression, but it did not directly predict them. The results implied that cognitive factors should be considered when examining the effects of media multitasking on anxiety and depression. We found that attention focusing and negative information attentional bias play serial mediating effects between media multitasking and anxiety/depression. In contrast, attention shifting did not play the same role as attention focusing. Specifically, individuals with more media multitasking behaviors have worse abilities in attention focusing, which will more frequently draw their attention to negative information, which then induces higher levels of anxiety and depression.

## Data availability statement

The raw data supporting the conclusions of this article will be made available by the authors, without undue reservation.

## Ethics statement

The studies involving human participants were reviewed and approved by the Ethics Committee of Tianjin Normal University. Written informed consent for participation was not required for this study in accordance with the national legislation and the institutional requirements.

## Author contributions

SL conceived the research idea and structured and drafted the manuscript. Both authors collected and analyzed the data, contributed to the article, and approved the submitted version.

## Footnotes

1
https://www.wenjuan.com

2
https://www.cpsbeijing.org/
